# The WOMEN-UP Solution, a Patient-Centered Innovative e-Health Tool for Pelvic Floor Muscle Training: Qualitative and Usability Study during Early-Stage Development

**DOI:** 10.3390/ijerph18157800

**Published:** 2021-07-23

**Authors:** Sònia Anglès-Acedo, Lorena López-Frías, Vicenç Soler, Joan Francesc Alonso, Arnoud W. Kastelein, Boris C. de Graaf, Eva. V. Vodegel, Jaana Tervo, Adriana Baban, Montserrat Espuña-Pons, on behalf of the WOMEN-UP Consortium

**Affiliations:** 1 Department of Obstetrics and Gynecology, Hospital Clinic de Barcelona, Universitat de Barcelona, 08036 Barcelona, Spain; llopez3@clinic.cat (L.L.-F.); mespuna@clinic.cat (M.E.-P.); 2 Department of Microelectronics and Electronic Systems, Universitat Autònoma de Barcelona, 08193 Bellaterra, Spain; vicenc.soler@uab.cat; 3 Department of Automatic Control, Biomedical Engineering Research Centre, Universitat Politècnica de Catalunya, 08028 Barcelona, Spain; joan.francesc.alonso@upc.edu; 4 Department of Obstetrics and Gynecology, Amsterdam UMC Location Academic Medical Center, 1105 AZ Amsterdam, The Netherlands; a.w.kastelein@amsterdamumc.nl (A.W.K.); borisc.degraaf@gmail.com (B.C.d.G.); e.v.vodegel@amsterdamumc.nl (E.V.V.); 5 Department of Physical and Rehabilitation Medicine, Kuopio University Hospital, 70210 Kuopio, Finland; jaana.tervo@kuh.fi; 6 Department of Psychology, Babes-Bolyai University of Cluj-Napoca, 400015 Cluj-Napoca, Romania; adrianababan@psychology.ro

**Keywords:** patient-centered innovation, qualitative study, usability study, pelvic floor muscle training, urinary incontinence, serious games, biofeedback, eHealth

## Abstract

e-Health may enhance self-management of pelvic floor muscle training (PFMT) to treat stress urinary incontinence (SUI). It is crucial to involve patients in planning, developing and monitoring the optimal e-Health solution. This research aims to describe patient-centered innovation in an early developmental stage of the WOMEN-UP solution. We conducted a qualitative study through a self-developed questionnaire in 22 women with SUI, to define system requirements from a patient’s perspective. The first prototype of the WOMEN-UP solution was developed. It was tested by 9 patients in a usability study (think-aloud protocol and retrospective interviews). Patient preferences regarding the possible use of an e-Health solution with serious games for PFMT were: (1) to receive feedback about PFMT; (2) convenient home-use; (3) increasing motivation; (4) available in medical centers. Identified usability aids (31) reassured our design-development plan, which considered the biofeedback and serious games as key factors. Patient’s perspective detected some unexpected issues related to the calibration and serious games, involving a change in the ongoing development to get an improved WOMEN-UP solution; the value of patient-centered innovation during the development of an e-Health solution for PFMT (WOMEN-UP solution). To identify patients’ unmet needs, we proposed a longitudinal approach for the future eHealth-related patient-centered innovations.

## 1. Introduction

It is estimated that one out of three women may be affected by pelvic floor dysfunction: urinary incontinence (UI), pelvic organ prolapse and anal incontinence [1]. Urinary incontinence can be experienced as a major burden on quality of life. Stress urinary incontinence (SUI), characterized by involuntary loss of urine during physical activity, coughing and sneezing, is the most common type (10–39%) [1]. Physical, sexual, social and emotional well-being can be disturbed in women with SUI.

Conservative management with lifestyle modifications and supervised pelvic floor muscle training (PFMT) is the recommended first-line treatment [2], especially in mild or moderate SUI. In standard care, this treatment usually involves regular visits to a clinic or hospital (e.g., 1/week) for a multidisciplinary approach provided by different clinicians (physiotherapists/physical therapists, nurses, midwifes or medical doctors).

e-Health has gained exponential popularity during recent years in urogynecology, specifically regarding pelvic floor muscle training (PFMT) for urinary incontinence (UI). Mobile applications, vaginal devices and telemedicine (remote monitoring, videoconferencing, phone call, asynchronous store and forward systems record) have been demonstrated as potential alternatives to usual care, with growing interest and availability in high-income countries [3]. During the COVID-19 pandemic, as patients were not allowed to attend to face-to-face interventions, such innovative ways to deliver healthcare have become the key for UI conservative management, to guarantee the first-line treatment according to guideline recommendations [3,4].

Innovation in medicine aims to find new alternatives to improve the health of patients. Nevertheless, new does not always mean improved. Some new practices are taken up and diffused throughout health systems [5], even when they are of limited benefit or unproven efficacy, or represent risks to patients, as many women’s experiences with transvaginal mesh for prolapse. Consequently, we should be cautious with innovation implementation, considering the possibility of both positive and negative impacts.

Focusing on conservative management for UI, e-Health may improve access to care. Often, women encounter difficulties to perform PFMT because of waiting lists, costs or limited access due to time constraints. Moreover, other frequently occurring barriers such as trouble remembering to exercise, doubts regarding PFMT performance and difficulty finding time [6] may be overcome by empowering self-management and improving patient adherence through an e-Health solution. However, it is unclear which patients are willing and able to accept e-Health. Some factors have been proposed as predictors of patient acceptance or resistance to consumer e-health services, including sociodemographic variables, device usability, awareness of the e-health innovations, and the user’s computer skills [7].

Implementation of eHealth interventions often fails, due to poor or lacking usability evaluation, which can lead to misuse of the intervention [8,9]. Therefore, new interventions must undergo a rigorous, suitable and user-based usability evaluation first [10,11,12]. Reliable and widely accepted usability testing methods, such as the think-aloud protocol and retrospective interviews, facilitate insight into usability issues (barriers) through observations of interface interaction [13]. Specifically, user-centered testing has been used for gamified health interventions [14] and biofeedback-assisted systems [15].

Therefore, to ensure if an e-health solution meets the needs of patients, it is crucial to involve patients in the development of the e-Health solution: patient-centered innovation. [16].

The WOMEN-UP solution is a patient-centered innovation developed from 2015 to 2019 in a project funded by the European Union’s Horizon 2020 research and innovation programme. The WOMEN-UP project aims to improve quality of life in women with SUI through an e-Health self-management solution for PFMT and lifestyle modification. In short, the WOMEN-UP solution consists of biofeedback devices (vaginal and abdominal probes) to record EMG data, a mobile application to make the user perform exercises using serious games (gamification) and a web platform that collects all data and enables personalized supervision and an engaging home-training environment. Biofeedback in PFMT, a direct audiovisual feedback system on pelvic floor muscle activity, can improve clinical outcomes [17]. The gamification of motor function rehabilitation may contribute to this physiotherapeutic intervention by improving user engagement and learning experience [18].

The WOMEN-UP consortium, composed of hardware and software engineers, psychologists and healthcare professionals, was guided by patient perspectives, in early- stage design as well as development of the WOMEN-UP solution. Accordingly, patients were involved in one of the 8 work packages to ensure patient-centered innovation.

The aim of this study was to describe patient-centered innovation in an early developmental stage of the WOMEN-UP solution, prior to future evaluation of functionality in a pilot study and future evaluation of efficacy in a randomized controlled trial.

## 2. Materials and Methods

The WOMEN-UP project was approved by ethical committees in all participating medical centers (HCB/2016/0480, NL56562.018.16, 324/13.02.00/2016). Informed consent was obtained from all subjects involved in the study.

In the present study, we focused on the first three stages of the project: qualitative assessment (stage 1), development of the first prototype (stage 2) and usability testing (stage 3).

### 2.1. Stage 1. Qualitative Assessment: Identify Key Stakeholders’ Needs

This task included identifying patients’ preferences for the conservative treatment of UI, aiming to define system requirements from a patient’s perspective. The WOMEN-UP consortium developed a set of 29 standard questions (Supplementary Material S1), open-ended and with answers on Likert scales. The questions covered issues like: global impressions about device functionality; physical characteristics of the device including materials, interface, batteries; instructions; serious games; feedback received (current and desired feedback, areas of improvement). Participants answered the questionnare after a self-experience with available commercial devices for PFMT: Neurotrack^®^ Myotrac Infinity Pro (2 Channels) (Thought Technology Ltd., Montreal, Quebec, Canada) and Femiscan^®^ (Mega Electronics, Kuopio, Finland). This information provided valuable input for the development of a new device.

#### 2.1.1. Participants

Women with mild or moderate SUI able to perform PFMT, who attended a urogynaecological department, were recruited in university hospitals in The Netherlands, Spain and Finland. Participants were invited for an intake visit in an outpatient clinic and were instructed to use the available commercial devices: Neurotrack^®^, Femiscan^®^ or both.

#### 2.1.2. Data Analysis

Quantitative data from questionnaires (numerical data, Likert-scale answers) were entered separately and analyzed with a quantitative analytic tool (SPSS 20, IBM, Armonk, NY, USA). Because of the low number of participants in each group, only basic descriptive analyses were conducted and reported (frequency of a particular answer and means together with SD).

As for qualitative data (answers in open-ended format), we analyzed and manually coded every interview form, according to the general themes covered by the questionnaire. Frequency analyses of answer categories (codes) were conducted.

### 2.2. Stage 2. WOMEN-UP Solution: First Prototype

**Figure 1:**
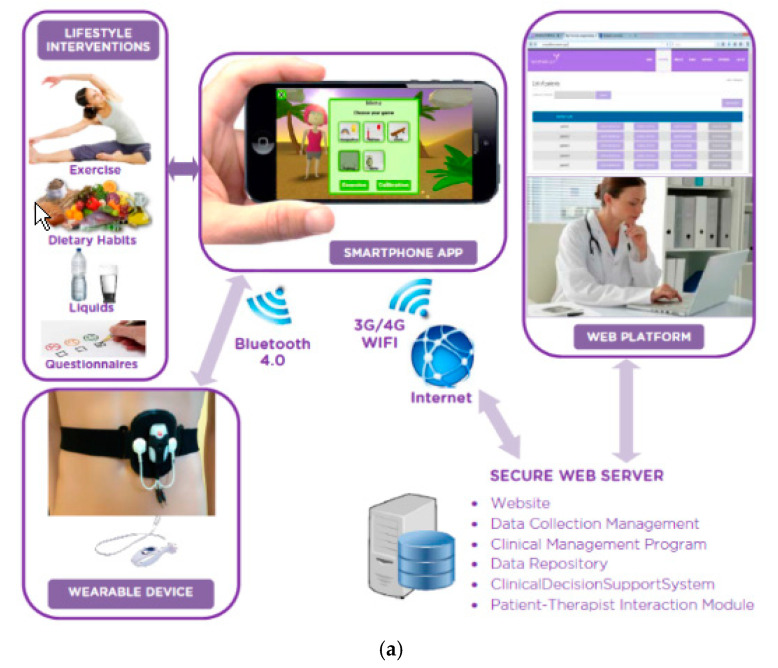

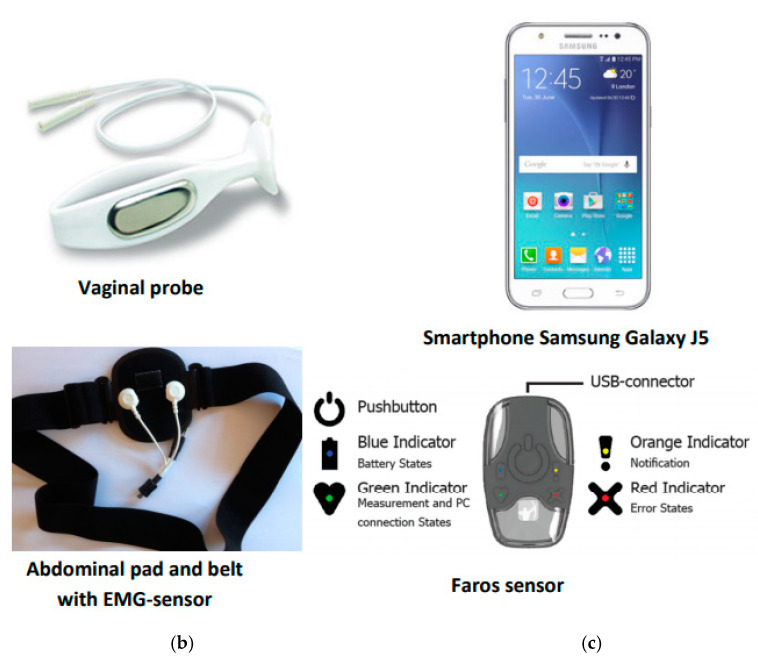
First prototype of the WOMEN-UP solution. (**a**) Elements of the WOMEN-UP intervention, (**b**) Vaginal biofeedback device (pelvic floor muscles), (**c**) Abdominal biofeedback device (rectus abdominis muscle).

The information provided in stage 1 was used to developed an early, operational prototype of the intervention. This system was comprised of a vaginal and abdominal biofeedback device, a serious gaming application for a smartphone, a therapist–patient web-based communication platform and a personalized training schedule. Figure 1 shows the components of the intervention and their integration.

The smartphone and the abdominal biofeedback device connect wirelessly via Bluetooth. Pending the development of a wireless vaginal device in a future stage of the project, this first prototype used a commercial vaginal probe, which was connected by wires to the abdominal device. The devices measured electromyographic activity of the pelvic floor and the abdominal muscles. Muscular activity recorded from both muscles will be compared to the exercise programmed by the therapist (in terms of achievement of the number of contractions, their duration and level, and the relaxation periods). The connection with the smartphone enabled the games to provide real-time biofeedback and detailed insight into the progress and results of the training.

The secure online platform, also under development during this study, facilitated written communication with the therapist. Both user and therapist had access to the details of the completed and planned exercises. Data protection regulations for security and privacy were considered carefully. All patient data were encrypted and stored in a database in the web platform server independently of personal data. Patient data recorded by the personal devices were encrypted with the SSL certificate (SHA-256) and stored securely hashed with an encryption algorithm. Communication between the smartphone and the web platform was secured with encrypted transmission via the HTTPS protocol.

### 2.3. Stage 3. Usability Testing

The first prototype developed in stage 2 was used for user-centered usability evaluation through a think-aloud method. It is vital that participants feel confident and comfortable with the think-aloud session and the investigators [19,20], so we decided that the investigators should be usability-trained healthcare professionals with expertise on PFMT. Additionally, the healthcare professional acted as spokesperson for usability issues and performed both think-aloud and retrospective interviews.

In phase one of the usability testing, a therapy simulation with elements of standard PFMT was performed in which participants tested the usability of the WOMEN-UP solution while thinking aloud with a healthcare provider.

In phase two of the usability testing, participants gained instructions to use the first prototype of the WOMEN-UP solution in a home setting for two weeks. After two weeks’ use, a second think-aloud testing and a retrospective semi-structured interview were performed.

One usability-trained healthcare professional conducted the interviews with all participants, guided by a retrospective questionnaire on usability, functionality, satisfaction and preference. The interviews and think-aloud sessions were audio-recorded and observational notes were taken. All audio-recorded data and written notes were transcribed verbatim and imported into MAXQDA software (VERBI Software, Berlin, Germany) (version 12.2.1).

#### 2.3.1. Participants

Volunteers were recruited in university hospitals in The Netherlands, Spain and Finland. Participants fluent in English were invited for an intake visit in an outpatient clinic and received instructions on PFMT and a brief introduction to the eHealth solution by a healthcare professional.

#### 2.3.2. Qualitative Analysis

Computer-assisted qualitative analysis was performed using a thematic label framework based on the heuristics for medical device evaluation [21]. After data familiarization and iterative coding in data of two participants, the framework was complemented with additional relevant labels of the relevant themes from the model for user acceptance of technology [22] and serious gaming components [14,18].

The data were consolidated through the deletion of duplicate coded text segments. The unique text segments were manually summarized into written positive usability comments relating to user experience and usability issues that violated prior determined heuristic themes. Text segments with positive usability comments were manually separated from the issues, summarized and categorized. Usability issues were sorted according to the place of occurrence in the WOMEN-UP solution and observation frequency among participants. Subsequently, all issues were scored according to the Nielsen severity scale. The scale ranges from 0 to 4, representing: nihil (0), trivial (1), minor (2), major (3) and severe (4) problems [23].

#### 2.3.3. Multidisciplinary Solution Optimization

Hardware and software engineers, psychologists and healthcare professionals combined their expertise in a multidisciplinary panel discussion. The usability aids and issues were discussed, and the consortium partners devised possible system improvements, in addition to already planned features and functionalities. The possible improvements were discussed after informal assessment of the state of development of system elements, the available resources, and the feasibility of the proposed improvements.

## 3. Results

### 3.1. Stage 1. Qualitative Assessment: Identify Key Stakeholders’ Needs

Overall, 22 patients—10 in the Netherlands (45.4%), 6 in Spain (27.3%), 6 in Finland (27.3%)—took part at this survey. Fourteen of these patients used both devices, whereas five used only Neurotrack^®^ and three only used Femiscan^®^. Due to the small sample sizes in different countries, we present the overall findings and will not make inter-country comparisons.

The mean age of participants was 47 years old (SD: 12 years, range 29–71 years). The mean number of vaginal deliveries was 1.85 (SD 1.22, range 0–5). The mean duration of UI was 59.23 months (SD: 151.69 months, range: 2–444 months). The medical supervision of the patients varied from 0.5 to 360 months (mean = 12.83, SD = 277.06). The majority of patients (59.1%) had not been treated before for UI. Those treated previously with PFMT reported being moderately satisfied with a mean satisfaction of 3.68 (SD = 1.11), on a scale varying from 1 to 5.

Nearly three out of four patients indicated that the e-Health solution should be available in medical centers. Table 1 shows the quantitative indicators for the pelvic floor muscle device features and integrated system. Overall, the device should be reliable and easy to use, and when it is used it should provide clear feedback on how the exercises are performed.

The qualitative indicators for pelvic floor muscle device features and integrated system are summarize below (Table 2).

The most common comments from patients’ perspectives were related to:

- Adherence improvement:
  “*Having the home device allows greater availability and gives them autonomy, which facilitates the adherence to the PFMT.*”
  “*The verbal and visual instructions facilitate the successful accomplishment of the PFMT, and consequently, improve concentration and adherence to treatment.*”
  *“Having a biofeedback device at home helps them to motivate themselves to continue doing the exercises, thus creating more adherence to PFMT.”*
  *“The possibility to train home increased the amount of exercises done during the study period.”*
- Performance improvement:
  *“A key issue was also the easy-to-use technique with easily found start-buttons and no wires and very clear instructions.”*
  *“Devices help to improve the self-awareness of pelvic floor muscles by vaginal probe and by control over the use of the abdominal muscles, and allow better integration about the instructions given by the therapist.”*
  *“Self-assessment in each session may increase the motivation to overcome the results of the training itself.”*

### 3.2. Stage 3. Usability Testing

Nine women, three in each center, volunteered to participate in the usability testing. Seven out of nine were healthy volunteers, two volunteers had complaints of SUI. The mean age was 41 years old (range: 25–61 years). Seven women were parous. Fifty-six percent were familiar with pelvic floor exercises. All participants completed the two weeks of training. Two out of nine women reported having limited experience with smartphone applications. The think-aloud interviews of the first phase failed in three participants due to the absence of the interviewer at the start of the study.

#### 3.2.1. Usability Aids

Analysis of labeled text segments showed 31 usability aids that appeared to positively affect user experience (Table 3). These predominantly concerned remarks on the games and direct visual feedback of muscular contractions. Participants expressed that those exercises were more fun than dry exercises, or even that exercises would not have been done at all without the WOMEN-UP solution. The interactive design and the challenge of games were appreciated by multiple participants. The direct visual feedback without time lag provided the participants with valuable information, because of the difficulty of dry exercises. Also, it was reported by users that these features contribute positively to performing PFMT.

#### 3.2.2. Usability Issues

**Figure 2:**
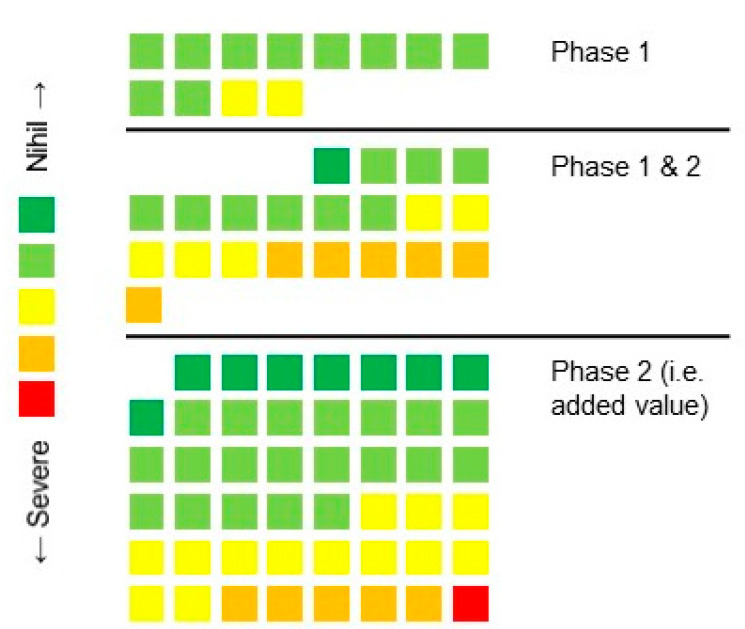
Colored blocks represent the usability issues with corresponding severity score. The order shows the value of each study phase.

Analysis of labeled text segments showed 80 usability issues. Figure 2 shows the role of the first and second phase of the usability study.

The non-persisting issues (12 issues only detected in phase 1) concerned 10 trivial issues and 2 minor issues (15% of all detected issues). These issues were most often related to either the calibration of the devices or the navigation of the mobile application such as simple navigation buttons. Among all identified issues in phase 1, 64% (21/33) were also identified in phase 2, meaning that most of the issues were persistent. On the other hand, newly identified issues in the second phase accounted for 59% (47/80) of all issues (Figure 2. Among them, the majority were nihil (17%), trivial (42%) or minor (28%) issues, although new major and severe issues were also identified (13%). These severe and major issues were related to communication, calibration and serious games.

These usability issues could predominantly be classified into 11 categories: mobile application general, mobile application navigation, calibration, serious games, gaming biofeedback, training evaluation, web platform, devices, device connectivity, training schedule, communications with the user. Among all identified issues, two-thirds are expected barriers due to the early-stage of development of the first prototype and all of them are already considered in the ongoing design and development of the WOMEN-UP prototype (e.g., device design and connectivity, communication with the user, web-platform and navigation app functionalities…). However, one-third of the detected issues were unexpected, mainly related to the calibration which required a new adjustment of the software or related to the serious games which required design of new type of games, as well as improvement of the patient’s manual or improvement of the feedback at the end of the performance (e.g., stars). Table 4 shows a summary of the usability issues.

#### 3.2.3. Multidisciplinary Solution Optimization

A list of improvements was formed by the team of hardware and software engineers, psychologists and healthcare professionals after a collaborative review of issues, review of proposed improvements and discussion on the impact and feasibility of these proposed improvements.

Afterwards, most issues were classified to ensure urgent implementation, and were implemented and solved during the next phase of the project: an evaluation of the functionality of the three components of the solution in a pilot study. Overall, urgent issues to be solved were identified in relation to the web platform, the mobile application and the serious games.

Few issues were classified as non-urgent, and were implemented and solved in a later phase, prior to evaluation of efficacy in a randomized controlled trial. Some of those issues were related to vaginal device design or device connectivity.

## 4. Discussion

The present study demonstrated the value of patient-centered innovation during the development of an e-Health solution for the conservative treatment of SUI (WOMEN-UP solution) in two different stages: (1) qualitative study, and (2) usability study.

### 4.1. Stage 1. Qualitative Assessment: Identify Key Stakeholders’ Needs

During an early-stage of the design (stage 1), we identified patients’ needs through a qualitative study.

We included women aged 29 to 71 years old, which is a good representation of the female population affected by SUI, permitting us to design an e-health solution suitable for a wide range of age.

According to the participants, the main benefits of using an e-Health solution with serious games for PFMT were: (1) to receive feedback about the performed pelvic floor contractions; (2) convenient home-use; (3) increasing motivation to perform the PFMT. These data were used by the WOMEN-UP consortium to design a web platform, serious games and a vaginal device.

Most participants reported they would like to receive feedback on performance as well as on progress and adjustment. This may explain why three-quarters of participants preferred to use the WOMEN-UP solution in a medical center. Therefore, such supervision by a healthcare professional is one of the key points in the design and development of the WOMEN-UP solution to provide a solution to a patient’s unmet needs. Supervised PFMT is also part of all international recommendations [2]

Definition of the new WOMEN-UP solution from patient’s point of view was considered as key information to understand how the first prototype should be built. According to the methods to achieve person-centered care proposed by Bhattacharyya et al. [24], we followed a need-focused approach (customers have unmet needs which are not well defined but could be met by new services with new processes that have yet to be developed), providing us with a clear definition of a patient’s needs and of a new solution. Afterwards, to best way to understand what patients really want is by rapidly building a prototype (stage 2) that has enough functionality for potential patients to use, and give feedback, which was done in stage 3 of the present study.

### 4.2. Stage 3. Usability Testing

According to the usability aids found in our study, the use of the WOMEN-UP solution provided autonomy, feedback on performance and made exercises more fun. These findings confirmed the findings from our previous qualitative study (stage 1) and demonstrated the importance of the serious games and the feedback devices within the WOMEN-UP system. In addition, it reassured our design-development plan, which considered the biofeedback and serious games as key factors. Accordingly, feedback was found to be one of the most important game elements in a systematic review focused on the effect of gamification [25].

The longitudinal design of the usability testing provided us with interesting information about patient’ needs in different moments. Phase 1 provided insight in the usability issues that participants experienced after first-time use and usability issues that positively influenced user experience. Phase 2 provided insight in usability issues that positively influenced user experience and usability issues that persisted to obstruct task performance after prolonged use at home, and potential new issues. Understanding patient perspectives is critical to developing tailored care approaches that address patients’ needs and wants, with the ultimate goal of improving patient satisfaction and quality of life [26].

Most identified usability issues were already planned to be solved in the ongoing design and development of the WOMEN-UP prototype, but such expected barriers confirmed the agreed design by the multidisciplinary team and reassured the investment plan of the resources. In addition, the patient-centered innovation assessment permitted us to identify some unexpected issues, which allowed us to implement new functionalities according to our patients’ needs. Therefore, most of the issues indicated by the study participants were implemented urgently to guarantee a new and improved WOMEN-UP solution (second prototype). This second prototype will be extensively tested for functionality of the three components in a pilot study (will be reported in a follow-on paper).

## 5. Conclusions

In conclusion, this qualitative study and usability testing generated important suggestions for the prototype development of an e-Health tool for PFMT. In the context of future eHealth-related patient-centered innovations, we demonstrated the added value of a longitudinal approach that involved patients in an early stage of the design and development.

## Figures and Tables

**Table 1:** Summary of quantitative indicators for the pelvic floor muscle device and the integrated system.

Feature	Option	Responses	% Participants
Vaginal probe shape	oval and conical with stop	11	50.0%
	cylindrical with stop	5	22.7%
	cylindrical without a stop	4	18.2%
	other shape with stop	2	9.1%
	Valid responses	22	100%
Vaginal probe material	smooth surface	19	86.4%
	neutral	18	81.8%
	soft	12	54.5%
	flexible	10	45.5%
	hard	9	40.9%
	rigid	9	40.9%
	warm	7	31.8%
	rugged	1	4.5%
	cold	1	4.5%
	Total responses	87	
Device perception	comfortable	18	81.8%
	easy to use	17	77.3%
	inexpensive	17	77.3%
	motivating	16	72.7%
	reliable	15	68.2%
	versatility	14	63.6%
	good design	12	54.5%
	Total responses	110	
Motivation to exercise	they help to increasemy motivation	18	81.8%
	they do not modifymy motivation	2	9.1%
	Valid responses	20	90.9%
	Empty responses	2	9.1%
Feedback preferences	any time needed	6	27.3%
	monthly	5	22.7%
	at the end of thetraining period	2	9.1%
	every 15 days	2	9.1%
	Valid responses	15	68.2%
	Empty responses	7	31.8%

Note that the participants were allowed to select more than one option regarding some of the features; in these cases, the percentages corresponding to valid and empty responses are not shown.

**Table 2:** Summary of qualitative indicators for the pelvic floor muscle device and the integrated system.

Topic	Recommendations	Risks
global functionality	convenient use at home valuable feedback enhanced motivation	finding the right time/place(tiredness, busy schedule) use difficulties too simple/not stimulating
mechanical design	oval/conical with stop several sizes light and smooth wireless simple interface (few buttons)	wrong size hard to reach buttons hard/cold materials rough materials
system features(in order ofimportance)	reliability ease of use motivating/entertaining clear feedback/guidance	too simple/not stimulating too difficult to use or follow
device instructions	clear with pictures/diagrams with safety/hygiene information describing the training program	too simple instructions too complex instructions too many program alternatives
feedback	exercises correctly performed information on strength summary after exercising improvement over time	ambiguous feedback monotonous feedback too complicated feedback
patient–professionalinteractionpreferences	necessary, even if minimal (messages after first visit) provide feedback onperformance adjust treatment	incorrect use if there is nointeraction poor adherence because of lack of interaction
system interface	smartphone or tablet-based providing feedback during andafter training clear instructions/guidance	not familiar withtechnology data safety
serious games	should enhance motivation training for 10 to 20 min	overtraining incorrect training

**Table 3:** Summary of usability aids.

Category	Summarized Facilitator	Description	Number of Facilitators
Mobile Application general	Positive attitude toward the system	Positive attitude towards the system, application and the flexibility provided by the solution	7
Calibration	Easy-to-learn calibration	The calibration of the devices has a fast learning curve	1
Serious games	Good biofeedback	Users appreciate the usefulness of direct visual feedback on the muscular contractions and the final scores provided	7
	Good control during games	Additional feedback given to the user (next actions to be performed, remaining time) helps the feeling of being in control of exercising	3
	Good game design	Games are well designed for these kind of rehabilitation exercises	1
	Games facilitate exercising	Exercises could not have been done without the games	4
	Game choice	Users can choose from different games, based on their preferences, to perform the same exercise	2
	Enjoyable gaming	Games make exercising fun and enjoyable	4
Devices	Functioning and comfort	Users feel comfortable with the use of the wearable device	2

**Table 4:** Summary of the usability issues (barriers).

Category	Issues	Type	Description	Comments
Mobile Application general	9	3 major, 2 minor 1 trivial and 3 nihil	Not all the functionalities were in the App, outdated design and sometimes used medical jargon abbreviations (PFMT)	As this is a pre-product test step, the online platform was chosen to summarize all functionalities since it was easier to increase the software functions without needing to install new App versions constantly. In addition, design was applied for functionality above cosmetics.
Mobile Application navigation	7	6 trivial and 1 minor	User could not use back button and the design (visibility) of some buttons could be improved.	Some issues were due to an incomplete development at the time of testing.
Calibration	9	4 trivial, 4 minor and 1 major.	The major issue was due to an absence of assistance/messages in case of error in the process. In addition, the display of the calibration process can be improved with more information and clear visibility along the whole process to guide the user.	Improvement implemented by a simpler calibration method.
Serious games	13	5 major, 3 minor and 5 trivial.	Some games and menus were unclear and with lags. Absence of encouragement and levels in some games. The visualization of some elements could be improved.	Major and minor issues information was used to help improving the application for future versions. The absence of description was improved by means of implementing a demo screen before playing,
Gaming biofeedback	6	2 minor and 4 trivial.	Most of the issues are referred to the score visualization, since it complicates the visualization of the game and it is uninformative. They also refer to the abdominal muscle activity: visualization and artifacts.	The score issues were improved by showing the information graphically and concentrated in the post-game screen. Respecting the abdominal muscle activity, instructions were added to explain to the user how to avoid artifacts.
Training evaluation	5	1 major, 2 minor and 2 trivial	Major and Minor issues referred to lack of feedback information after performing the exercises and also on how to perform the exercises (relaxation and contraction).	The online platform contains the feedback information since it should also be accessible by the therapist, as mentioned in the Mobile Application General section. Other issues were used to help improving the application.
Web platform	4	1 major and 4 trivial.	The major issue is referring to the poor visualization of the results. Trivial issues refer to some issues on the information display.	In the major issue, the visualization was more intended to be useful for the study outcomes. The other issues were used to help improve the web platform.
Devices	12	1 minor, 9 trivial and 2 nihil.	Most of the issues referred to the feeling of the devices. Vaginal: unpleasant, rigid, lack of low-battery warning, only 1 size; abdominal: narrow belt, hook-in system. Also lack of available instructions.	In most cases, the issues referred to the design, which maintained unaltered for measurability reasons.
Device connectivity	4	1 major, 2 minor and 1 trivial	Lack of assistance and messages when connecting or having troubles connecting. Also, names of the devices were unclear.	Improvements implemented through the manual, increasing the visual pop-up, when possible. Issues concerning Android were unaltered.
Training schedule	4	1 minor, 2 trivial and 1 nihil.	In general, the user was confused with the names given to the training sessions when having to select to recover not-performed sessions. They were marked as uncompleted and the user was disappointed with such labels.	In general, they were expected issues due to an incomplete development at time of testing.
Communications with the user	7	1 severe, 1 minor, 2 trivial and 3 nihil.	User reported some errors concerning not being aware of receiving messages since they were shown at the moment the user uses the App, such as communications of the therapist, reminders (questionnaires, exercises…), etc. Other reported issues were the web accessibility of the instructions (only on paper).	Some of them were expected since uncomplete development at time of testing and were improved later on.

## Data Availability

The data presented in this study are available on request from the corresponding author. The data are not publicly available due to privacy reasons.

## Supplementary Materials

The following are available online at https://www.mdpi.com/article/10.3390/ijerph18157800/s1, S1: The WOMEN-UP consortium developed a set of 29 standard questions during the qualitative study (stage 1).
