# Supplementary material for: The WOMEN-UP Solution, a Patient-Centered Innovative e-Health Tool for Pelvic Floor Muscle Training: Qualitative and Usability Study during Early-Stage Development
Source: Int J Environ Res Public Health. 2021 Jul 23;18(15):7800. doi: 10.3390/ijerph18157800 (PMC8345479; doi:10.3390/ijerph18157800)
Supplement: Supplementary file 1 [file ijerph-18-07800-s001.zip › ijerph-1293238-supplementary.pdf]

Annex 1. Questionnaire administered to patients

PARTICIPANT INFORMATION SHEET:

INVITATION TO PARTICIPATE:

Dear Participant:
As a woman aged 18 or over, who suffers from urinary incontinence, you are invited to participate in a written interview in the framework of the study entitled:

COST EFFECTIVE SELF-MANAGEMENT OF URINARY INCONTINENCE ADDRESSED TO WOMEN ACROSS EUROPE

ORGANIZED by: European Commission (H2020)

ACRONYM: WOMEN-UP

GRANT AGREEMENT NUMBER: 643535

Before you confirm your participation in this interview for this investigation study, it is important that you know what it consists of. Please read this document carefully:

STUDY OBJECTIVES: The main objective of WOMEN-UP project is to improve the quality of life of urinary incontinence patients by developing a new technology that allows for the self-management of the chronic UI disease via a device for pelvic floor muscle training connected to a platform with a decision support system and secure remote medical supervision.
The specific objective of the interview you would be taking part in is to get a clear picture of the women's needs and opinions regarding home pelvic floor muscle training in order to build the new system. You may be asked by your health care provider to test at home two commercial devices for pelvic floor muscle training for a week prior to completing the interview.

VOLUNTARY PARTICIPATION: You are completely free to choose to participate in the study filling a written interview or not and to quit at any time.

NUMBER OF WOMEN AND ESTIMATED PARTICIPATION TIME: this interview will be filled in by 21 women in three European countries. The estimated time to fill in the interview is one hour.

STUDY PROCEDURES: Once you have read this information sheet and signed the annexed informed consent form, your participation will consist on using two commercial devices for home pelvic floor training following your doctor recommendations and/or completing a written interview about the use of devices for pelvic floor muscle training

EXPECTED BENEFITS AND RISKS: You yourself will not receive any direct benefit, but this interview will provide the investigators with a good understanding of the patient's needs and opinions regarding home pelvic floor muscle training in order to build and evaluate the new system. The interview doesn't involve risk or harm for participants. However, you can stop any time the interview.

HOW THE FINDINGS WILL BE USED:

The results of the study will be used for to improve the design of the device and supportive software for self-management of urinary incontinence, and for scholarly purposes. The results from the study might be published in a professional journal in the field of urogynecology.

CONFIDENTIALITY: a specific paragraph for data protection law in every country.

The handling, communication and transfer of all participants' personal data will be in line with that laid out in the Personal Data Protection Law 15/1999 of 13th December. The information collected will be assigned a code to prevent identification. The results of the study will be collected and stored anonymously and separately. When publishing the results no personal information will be made public that could identify you. Moreover, in accordance with the law, you have the right of ***access, modification, opposition and***

cancellation of the information *which can be exercised simply by getting in contact with the principal investigator at your centre who will inform the sponsor.*

If you have any doubts please contact: country specific contact address

Many thanks for your kind collaboration

INFORMED CONSENT

Title: COST EFFECTIVE SELF-MANAGEMENT OF URINARY INCONTINENCE ADDRESSED TO WOMEN ACROSS EUROPE

Grant Agreement number: 643535

I,…………………………………………………………………………… (Full name)

I have been informed about this study and have been able to ask questions about it to the extent that I feel that I have received sufficient information as to be able to participate in the investigation.

I have spoken with………………………………………..…………… (Interviewer's name)

I understand that my participation in the study is voluntary, that all personal information will be dealt with in ***strictest confidentiality*** *and that I can withdraw from the study:*

Whenever I want to

Without having to give any explanations

I hereby freely agree to participate in the study

Participant's signature ____________________ *Date: |____|_____|*

Day Month Year

*Interviewer's signature*____________________ *Date: |___||___|_____|*

Introduction

STUDY OBJECTIVES: The main objective of WOMEN-UP project is to improve the quality of life of urinary incontinence patients by developing a new technology that allows for the self-management of the chronic UI disease via a device for pelvic floor muscle training connected to a platform with a decision support system and secure remote medical supervision.

The specific objective of the interview you are taking part in is to get a clear picture of the women's needs and opinions regarding home pelvic

floor muscle training in order to build the new system.

Your participation will consist on completing a written interview about the use of devices for pelvic floor muscle training. The information collected will be assigned a code to prevent identification. The results of the study will be collected and stored anonymously and separately. When publishing the results no personal information will be made public that could identify you.

The interview is divided in two sections: a) socio demographic data b) open questions and questions with specific response options.

Please, answer according to your personal opinion. You could comment any aspect that you deem necessary. Do not worry if yo u repeat information considered important in different questions.

THANK YOU

Socio demographic data

Fill in the following socio-demographic and clinical questions, which are going to be used only for descriptive purposes.

- *BIRTHDATE (mm/dd/yyyy):*
- *WEIGHT(Kg):*
- *HEIGHT(m):*
- *TYPE OF URINARY INCONTINENCE:*

STRESS INCONTINENCE

URGE INCONTINENCE

MIXED INCONTINTENCE

OTHER:_____________

- *NAME OF THE DEVICE/DEVICES USED*

NAME: Neurotrac *For how long have you used the device?: ___________*

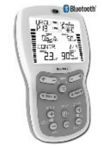

NAME: Femiscan *For how long have you used the device?:___________*

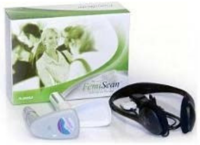

OTHERS: ________ *For how long have you used the device?:___________*

NONE

- *DURATION OF THE INCONTINENCE (ex. 4 months, 2 years…)*
- *SEVERITY OF THE INCONTINENCE* *VERY MILD*

MILD

MODERATE

SEVERE

- *TIME UNDER MEDICAL SUPERVISION:_____________*
- *HAVE YOU USED PREVIOUS TREATMENTS FOR UI?*

☐ *NO*

☐ *YES*

*IF YOU MARKED **YES,** PLEASE TICK BELOW THE ONE(S) YOU USED AND HOW EFFECTIVE/SATISFIED YOU ARE WITH THEM*	*How effective/satisfied*				
	1	*2*	*3*	*4*	*5*
☐ *PELVIC FLOOR MUSCLE TRAINING*	*1*	*2*	*3*	*4*	*5*
☐ *PESSARY*	*1*	*2*	*3*	*4*	*5*
☐ *MEDICATION*	*1*	*2*	*3*	*4*	*5*
☐ *SURGERY*	*1*	*2*	*3*	*4*	*5*
☐ *OTHERS:*					

- *TOTAL NUMBER OF DELIVERIES:* ☐
- *TOTAL NUMBER OF VAGINAL DELIVERIES:* ☐

SECTION 2

1. From your point of view, what are the main benefits of using a device for pelvic floor muscle training?

2. And what are the main disadvantages and concerns about the use of a device for pelvic floor muscle training?

3. Considering your recent experience with biofeedback devices provided for this study, how would your perfect device be? (Kind of instructions/information, size, shape…)

4. What do you think of the probe you have to introduce in the vagina, from the perspective of the devices you used?

4a. What would the best shape be?

OVAL AND CONICAL WITH "STOP"	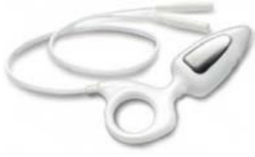 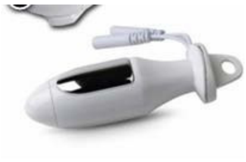
CYLINDRICAL WITH STOP	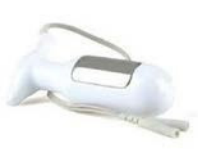 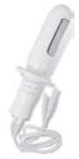
CYLINDRICAL WITHOUT A STOP	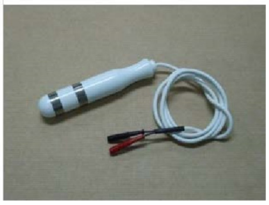 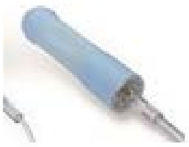
OTHER SHAPES WITH STOP	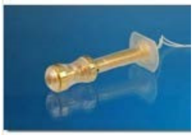 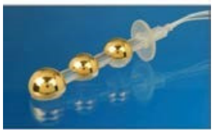
OTHER SHAPES WITHOUT STOP	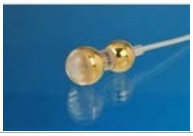

4b. What would the best size be?

4c. What would the best properties of material be? (you can choose more than one option)

☐ *Hard*	☐ *Soft*	
☐ *Rigid*	☐ *Flexible*	
☐ *Warm*	☐ *neutral*	☐ *cold*
☐ *Rugged*	☐ *smooth surface*	

5. What are the things (physical characteristics) that you don't particularly like of the devices you have used? / What would you change?

- *About neurotrac:*

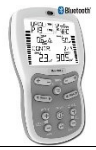	

- *About Femiscan:*

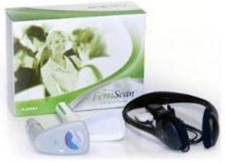	

- *Any other device you are familiar with:*

Any other device you have used (please write the name if you remember it)	

6. Which device from the above do you prefer?

   - ☐ Neurotrac
   - ☐ Femiscan
   - ☐ Other device. Name ____________________

Comments:

7. What do you think of the automatic programs the devices usually offer? For instance regarding program offer (too few, too many, just the right number), complexity (easy, too difficult, just right)….or other things that you have in mind?

8. What do you think of the autonomy/way of charging the battery of the device(s) you have used? (you can choose more than one option)

   - ☐ *Battery life too short*
   - ☐ *Battery life long enough*
   - ☐ *Difficult to charge*
   - ☐ *Easy to charge*
   - ☐ *Other:* ________

9. What would you think of using a smartphone or a tablet as an interface to the pelvic floor muscle training device (the smartphone would be thus the tool to communicate with and receive info from the device)?

Introduction: Some medical devices are accompanied by games related to physical training that patient can do while engaging in their rehabilitation exercises or simply in physical activity (ex. Nintendo Wii). Related to pelvic floor training…

10. To what extent do you think a game like this would enhance your motivation to start pelvic floor training/to stick to the pelvic floor training program? Why?

11. Have you played this sort of games before? (not necessarily related to pelvic floor training but also to other physical activities/rehabilitation exercises) If yes, how often? Can you recall some of their names?

12. What were the games you particularly liked? Why?

Introduction: as you know, the devices give you information (feedback) on how to perform the exercises, and have memory that records your progress....

13. What kind of information/feedback would you like to have while you do the exercises?

14. What kind of information/feedback would you like to have right after you do the exercises? Prompts: reference to a mean/standard performance, reference to my previous performance, breakdown of feedback (contraction time and contraction strength), general motivating comments etc.

15. What would you change to improve the feedback of the device(s) you used?

16. Do you think obtaining information/feedback from the device alone is enough, or would you rather have some sort of input from your health care provider?

17. Which health care provider would be more suitable for this role? (you can choose more than one option)

- ☐ *Doctor*
- ☐ *Nurse*
- ☐ *Physiotherapist*

18. Imagine that the information recorded by the device could be sent to your health care provider, so they can track your progress:

18a. What kind of feedback would you like to have from your health care provider regarding your PF exercises?

18b. How often ideally?

- ☐ *Weekly*
- ☐ *monthly*
- ☐ *Anytime if the health care provider thinks there is something that I should know*
- ☐ *at the end of the prescribed training period other (please specify)*____

----

19. How do you think the use of these devices impacts your motivation to start the exercises routine? (you can choose more than one option)

- ☐ *They help to increase my motivation*
- ☐ *They do not modify my motivation*
- ☐ *I feel discouraged*

☐ *Other comments:* ____

20. How do you think the use of these devices impacts your motivation to maintain the exercises routine? (you can choose more than one option)

☐ *They help to increase my motivation*

☐ *They do not modify my motivation*

☐ *I feel discouraged*

☐ *Other comments:* ____

21. What conditions must be met to perform your exercises at home? (for instance, being alone at home, being alone in the room…)

22. What situations would prevent you more likely from training as you intended?

23. What kind of store do you think is more suitable to buy this device? (you can choose more than one option)

☐ supermarkets

☐ pharmacies

☐ through the medical centre

☐ internet Why?

24. What would be a fair price when the device is reimbursed by the patient’s insurance?

- ☐ *Difficult to tell*
- ☐ *Wouldn’t matter, it’s insured anyway*
- ☐ *50-100*
- ☐ *100-200*
- ☐ *200-400*
- ☐ *400-600*
- ☐ *600-800*
- ☐ *800-1000*
- ☐ *> 1000*

25. What would be a fair price when patients have to pay themselves for the device (out-of-pocket)?

- ☐ *Difficult to tell*
- ☐ *50-100*
- ☐ *100-200*
- ☐ *200-400*
- ☐ *400-600*
- ☐ *600-800*
- ☐ *800-1000*
- ☐ *> 1000*

26. Would you consider using a (completely refurbished and tested) device that has been used previously, if this would substantially reduce the price?

27. How did you feel when you first used a device for pelvic floor training?

28. Would you recommend the use of a pelvic floor training device to others that have your same problem? And why?

29. In your opinion, what would be a central feature the manufacturer would have to make sure to keep in a pelvic floor training device?

We would like to know how important the following characteristics of a pelvic floor training device are for you.

This scale is numbered from 0 to 100.

100 means the most important thing to consider, 0 that the characteristic is completely dispensable.

Draw a line from each characteristic to the point of the scale that better describes the importance it has for you. You can locate several characteristics at the same level

Please write the number you marked on the scale in the box below

CHARACTERISTICS	SCORE
Reliable/I get the feedback I need	
Comfortable to use	
Motivating	
Design/looks good	
Inexpensive	
Easy to use	
Versatility to use in different conditions/locations	

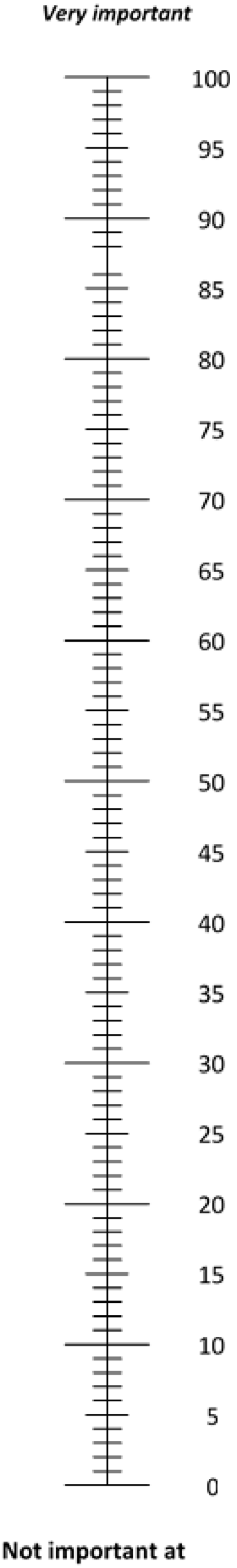

THANK YOU
